# Association between sleep duration on workdays and blood pressure in non-overweight/obese population in NHANES: a public database research

**DOI:** 10.1038/s41598-022-05124-y

**Published:** 2022-01-21

**Authors:** Yingjie Su, Changluo Li, Yong Long, Liudang He, Ning Ding

**Affiliations:** Department of Emergency Medicine, The Affiliated Changsha Central Hospital, Hengyang Medical School, University of South China, No. 161 Shaoshan South Road, Changsha, 410004 Hunan China

**Keywords:** Cardiology, Health care

## Abstract

This study aimed to explore the association between sleep duration on workdays and blood pressure (BP) including systolic blood pressure (SBP) and diastolic blood pressure (DBP) in non-overweight/obese population. A cross-sectional study composed of 2887 individuals from NHANES was conducted. Subjective sleep duration on workdays were evaluated by the questionnaire. Multiple linear regression analyses were done to explore the relationship between sleep duration and BP. Compared with sleep duration of 6–8 h, both sleep duration < 6 h and ≥ 8 h on workdays were significantly associated with increased SBP (β, 3.58 [95% CI 1.60, 5.56] and 1.70 [95% CI 0.76, 2.64], respectively). However, the significant association was not founded in DBP. The stratified analyses showed that in females, sleep duration (< 6 h or ≥ 8 h) on workdays were associated with SBP (β = 5.99 and 2.41, respectively, both *P* < 0.0005). In addition, the SBP levels were much higher among participants aged (≥ 60) with sleep duration < 6 h. The effect size was 7.23 (*P* = 0.0217). In the subgroup classified by race, a significantly positive association between sleep duration (< 6 h, ≥ 8 h) and SBP can be seen in the White population (β = 6.64 (*P* = 0.0007) and 1.91 (*P* = 0.0215), respectively). In non-overweight/obese population, both short sleep duration (< 6 h) and long sleep duration (≥ 8 h) on workdays were correlated with higher level of SBP.

## Introduction

Hypertension is a global health challenge, caused by the integration and accumulation of environmental and genetic risk factors^1^. In the United States, approximately one-third of adults suffer from hypertension^2^. Despite the fact that the public's attention and treatment of hypertension has improved, hypertension no longer remains well-managed^3–5^. According to recent literature, it has been calculated that during 2015, systolic blood pressure (SBP) of at least 110–115 mmHg was correlated with greater than 10 million deaths and over 211 million disability adjusted life year(DALYS)^6^.

In recent years, the adjustment of lifestyle as the main means to prevent hypertension has attracted people’s attention. Sleep, as a critical role in cardiovascular fitness, has been identified as a critical life-style risk element for cardiovascular disease^7^. Sleep duration, particularly lack of sleep, could affect blood pressure (BP) through disturbances in autonomic and hormone balances, and also lead to increased obesity and metabolic disorders, and circadian rhythm disorders^8^. Meta-analysis showed that short sleep time was associated with an increased risk of hypertension^9^. Growing evidence has showed that BP is influenced by sleep duration^10–16^, which is based on different age, genders, and races to explore the association.

However, few studies have been performed to investigate the relationship between sleep duration and BP in people with different body mass indexes (BMI). Previous research has identified that people who are overweight and obesity were both strong influence factors on BP^17,18^. So far, few studies have been done to explore the possible relationship between sleep duration and elements of BP in people who are not overweight or obese. Hence, this study aimed to determine the association between sleep duration on workdays and BP in non-overweight/obese population.

## Methods

### Study population

The National Health and Nutrition Examination Survey (NHANES) is the only national survey that offers a cross-sectional view of nutrition and health in the United States population. It collects information about demographics, health and health behaviors. Data researchers and users can use the survey data of the NHANES on the Internet. Details statistics of NHANES can be found on www.cdc.gov/nchs/nhanes/. All methods in our research were performed in accordance with the Declaration of Helsinki.

**Figure 1:**
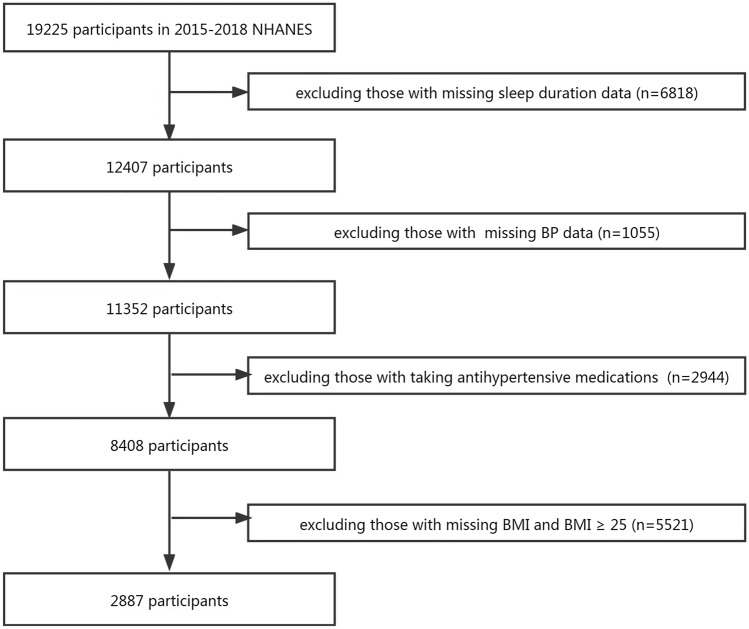
Flow chart and study design. *BP* blood pressure, *BMI* body mass index.

According to WHO guidelines, BMI is divided into underweight (< 18.5 kg/m^2^), normal weight (18.5–24.99 kg/m^2^), overweight (25–29.99 kg/m^2^) and obesity (≥ 30 kg/m^2^)^19^. Non-overweight/obese is defined as people with BMI < 25. This research combined 2015–2018 data for analysis. A total of 19,225 potentially participants were enrolled, 16,338 participants were excluded for the following reasons: missing sleep duration data(n = 6818), missing BP data(n = 1055), taking antihypertensive medications(n = 2944), missing BMI and BMI ≥ 25(n = 5521). Finally, 2887 participants were included in the study (Fig. 1).

### Definition

Sleep duration on workdays was evaluated by the questionnaire with the following questions: “Number of hours usually sleep on weekdays or workdays”. Sleep duration was divided into three groups, which were < 6 h, 6–8 h, ≥ 8 h respectively, of which 6–8 h was used as the reference group.

The trained and certified examiners used the standardized protocols and calibrated equipment to get the blood pressure readings. Three consecutive BP readings were acquired via ausculatory means. If a BP measurement was not successfully completed, a fourth measurement was implemented. The average of all available measures was used.

### Covariates

Race was divided into four groups: Mexican American, white, black and other race. Alcohol consumption was defined as the response to the question:“In the past 12 months, how often did you drink any type of alcoholic beverage?”, the responses was classified into three groups: drinking, no drinking, not recorded. Smoking was defined as the response to the question:“Do you now smoke cigarettes?”, the responses was classified into three groups: smoking, not smoking, not recorded. Diabetes was defined as the responses to the question:“Have you ever been told by a doctor or health professional that you have diabetes or sugar diabetes?”, the responses was classified into four group: yes, no, borderline, not recorded. Hypertension was defined as the response to the question: “Have you ever been told by a doctor or other health professional that you had hypertension, also called high blood pressure?”. The response was classified into three group: yes, no, and not recorded. The snort or stop breathing was defined as the response to the question: “In the past 12 months, how often did you snort, gasp, or stop breathing while you were asleep?”. The answers were classified into three group: yes, no, and not recorded. The method of obtaining other covariates, include gender, age, albumin, creatinine, hemoglobin, total cholesterol (TC), aspertate aminotransferase (AST), high-density lipoprotein (HDL), body mass index (BMI), can be found at www.cdc.gov/nchs/nhanes/. Among this covariates, age, albumin, creatinine, hemoglobin, TC, AST, HDL, BMI as continuous variables. Gender, alcohol consumption, diabetes, smoking, race, hypertension, snort or stop breathing as categorical variables.

### Statistical analysis

All estimates were calculated accounting for NHANES sample weights. A weighted multiple linear regression model was used to assess the correlation between sleep duration on workdays and BP including systolic blood pressure(SBP) and diastolic blood pressure(DBP). The covariates mentioned above were adjusted as potential effect modifiers. The mean ± S.D and percentage were used to represent continuous variables and categorical variables, respectively. To calculate the differences between males and females, weighted linear regression models were used for continuous variables or weighted chi-square tests for categorical variables. The values of missing continuous covariates were indicated by dummy variables, including albumin, hemoglobin, creatinine and TC, AST, HDL and the missing ratios were 7.8%, 6.3%, 7.8%, 7.7%, 8.0%, 7.7% respectively. The missing categorical variables were included in the analysis as a single group. The statistical software packages R (http://www.R-project.org) and EmpowerStats (http://www.empowerstats.com) were used for the data analyses. When the *P* value was < 0.05, it was considered statistically significant.

### Ethics approval and consent to participate

The ethics review board of the National Center for Health Statistics approved all NHANES protocols. Informed consent was obtained from all subjects and/or their legal guardian(s).

## Results

### Participant characteristics and univariate analysis

Table 1 showed the description of weighted sociodemographic and baseline characteristics. In the study, 2887 participants were subclassified based on gender. Among the participants, the proportion of males and females were 44.81% (n = 1378) and 55.19% (n = 1509), respectively. With ethnicity, the proportion of Mexican American, White and Black were 6.27%, 64.62% and 9.94%, respectively. Overall, the mean (SD) values for age, albumin, creatinine, SBP, DBP, hemoglobin, TC, AST, HDL, BMI were 38.54 (17.72) years, 43.48 (3.58) g/L, 73.21 (19.73) umol/L, 115.33 (14.56) mmHg, 68.47 (10.08) mmHg, 14.07(1.44)g/dL, 4.67 (1.01) mmol/L, 23.18 (13.01) IU/L, 1.59 (0.43) mmol/L, and 21.97(2.06) kg/m^2^, respectively. Among the participants, 66.01% were consumed alcohol drinkers, 2.61% were diabetes, 3.16% were hypertension, 6.72% have snort or stop breathing. 17.99% were smokers. Sleep duration was divided into three groups, which were < 6 h, 6–8 h, ≥ 8 h, each with a proportion of 6.26%, 40.53%, 53.21%, respectively. The univariate analysis of potential confounding factors is shown in Table 2.

**Table 1:** General characteristics of 2887 participants included in the present study.

	Total(n = 2887)	Male(n = 1378)	Female(n = 1509)	*P* value
Age(years)	38.54 ± 17.72	37.15 ± 17.89	39.68 ± 17.50	0.0001
Albumin(g/L)	43.48 ± 3.58	44.57 ± 3.55	42.60 ± 3.35	< 0.0001
Creatinine(umol/L)	73.21 ± 19.73	83.91 ± 15.74	64.56 ± 18.34	< 0.0001
SBP (mmHg)	115.33 ± 14.56	118.08 ± 13.48	113.10 ± 15.01	< 0.0001
DBP(mmHg)	68.47 ± 10.08	69.46 ± 10.68	67.66 ± 9.49	< 0.0001
Hemoglobin (g/dL)	14.07 ± 1.44	15.04 ± 1.16	13.29 ± 1.14	< 0.0001
TC (mmol/L)	4.67 ± 1.01	4.51 ± 0.96	4.80 ± 1.03	< 0.0001
AST(IU/L)	23.18 ± 13.01	25.30 ± 15.67	21.47 ± 10.05	< 0.0001
HDL (mmol/L)	1.59 ± 0.43	1.43 ± 0.37	1.73 ± 0.43	< 0.0001
BMI(kg/m^2^)	21.97 ± 2.06	22.13 ± 2.06	21.85 ± 2.06	0.0004
**Alcohol consumption (%)**				0.2788
No drinking	9.22	9.96	8.62	
Drinking	66.01	66.35	65.73	
Not recorded	24.77	23.68	25.65	
**Diabetes (%)**				< 0.0001
Yes	2.61	4.06	1.44	
No	95.97	94.58	97.10	
Borderline	1.42	1.37	1.46	
**Hypertension (%)**				< 0.0001
Yes	3.16	4.58	2.00	
No	46.80	44.38	48.76	
Not recorded	50.04	51.03	49.24	
**Snort or stop breathing**				0.0002
Yes	6.72	39.08	44.22	
No	41.91	8.56	5.22	
Not recorded	51.37	52.36	50.56	
**Smoking (%)**				< 0.0001
No smoking	15.48	18.19	13.28	
Smoking	17.99	24.12	13.01	
Not recorded	66.53	57.69	73.72	
**Race (%)**				0.0001
Mexican American	6.27	6.45	6.12	
White	64.62	64.04	65.08	
Black	9.94	12.47	7.89	
Others	19.17	17.04	20.91	
**Sleep duration (%)**				0.0007
6–8 h	40.53	42.20	39.17	
< 6 h	6.26	7.77	5.04	
≥ 8 h	53.21	50.03	55.79	

Mean +/− SD for: continuous variables. *P* value was calculated by weighted linear regression model. % for: categorical variables *P* value was calculated by weighted chi-square test.

*SBP* systolic blood pressure, *DBP* diastolic blood pressure, *TC* total cholesterol, *BMI* body mass index, *AST* aspertate aminotransferase, *HDL* high-density lipoprotein.

**Table 2:** Univariate analysis for SBP and DBP.

Covariate	SBP (β, 95% CI, P)	DBP (β, 95% CI, P)
**Gender**		
Male	Ref	Ref
Female	− 4.99 (− 6.04, − 3.93) < 0.0001	− 1.80 (− 2.53, − 1.06) < 0.0001
Age(years)	0.37 (0.34, 0.40) < 0.0001	0.13 (0.11, 0.15) < 0.0001
**Race**		
Mexican American	Ref	Ref
White	2.47 (0.25, 4.68) 0.0291	1.93 (0.39, 3.46) 0.0142
Black	5.77 (3.07, 8.47) < 0.0001	2.07 (0.20, 3.95) 0.0303
Others	1.46 (− 0.98, 3.89) 0.2415	2.40 (0.71, 4.09) 0.0055
Albumin(g/L)	− 0.28 (− 0.44, − 0.13) 0.0002	− 0.11 (− 0.22, − 0.01) 0.0344
Creatinine(umol/L)	0.12 (0.09, 0.14) < 0.0001	0.04 (0.02, 0.06) < 0.0001
Hemoglobin (g/dL)	0.74 (0.36, 1.12) 0.0001	0.86 (0.60, 1.12) < 0.0001
TC(mmol/L)	3.21 (2.68, 3.74) < 0.0001	1.71 (1.34, 2.08) < 0.0001
AST(IU/L)	0.10 (0.06, 0.14) < 0.0001	0.04 (0.01, 0.07) 0.0060
HDL(mmol/L)	1.81 (0.55, 3.07) 0.0048	1.03 (0.16, 1.91) 0.0209
BMI (kg/m^2^)	1.03 (0.77, 1.28) < 0.0001	0.39 (0.22, 0.57) < 0.0001
**Alcohol**		
No drinking	Ref	Ref
Drinking	− 5.00 (− 6.85, − 3.15) < 0.0001	− 1.24 (− 2.52, 0.05) 0.0592
Not recorded	− 7.89 (− 9.92, − 5.86) < 0.0001	− 4.66 (− 6.06, − 3.25) < 0.0001
**Diabetes**		
Yes	Ref	Ref
No	− 8.75 (− 12.06, − 5.43) < 0.0001	− 0.91 (− 3.23, 1.40) 0.4383
Borderline	− 3.99 (− 9.51, 1.52) 0.1559	0.55 (− 3.29, 4.39) 0.7787
**Smoking**		
Smoking	Ref	Ref
No smoking	1.89 (0.09, 3.70) 0.0400	− 0.17 (− 1.43, 1.10) 0.7964
Not recorded	− 5.13 (− 6.52, − 3.75) < 0.0001	− 3.03 (− 4.00, − 2.06) < 0.0001
**Hypertension**		
Yes	Ref	Ref
No	− 16.43 (− 19.46, − 13.40) < 0.0001	− 10.14 (− 12.24, − 8.04) < 0.0001
Not recorded	− 15.47 (− 18.49, − 12.44) < 0.0001	− 10.79 (− 12.89, − 8.69) < 0.0001
**Snort or stop breathing**		
No	Ref	Ref
Yes	4.61 (2.40, 6.81) < 0.0001	1.00 (− 0.53, 2.52) 0.1998
Not recorded	0.67 (− 0.43, 1.77) 0.2345	− 1.21 (− 1.98, − 0.45) 0.0019

*CI* confidence interval, *Ref* reference, *TC* total cholesterol, *BMI* body mass index, *AST* aspertate aminotransferase, *HDL* high-density lipoprotein, *SBP* systolic blood pressure, *DBP* diastolic blood pressure.

### Association between sleep duration on workdays and blood pressure

The results of multiple linear regression analysis used to explore the relationship between sleep duration and SBP were shown in Table 3. In the crude model, the sleep duration with 6–8 h was compared as the control group. Sleep duration < 6 h was significantly positively correlated with SBP (β, 6.15[95% CI 3.88, 8.42]). However, a significant relationship was not found between sleep duration ≥ 8 h and SBP. After adjustment for gender, age, race (model I), we can observe a significantly positive association between sleep duration and SBP, the effect size of the group < 6 h and ≥ 8 h were (β, 4.17 [95% CI 2.19, 6.15]), (β, 1.55 [95% CI 0.60, 2.51]), respectively. Similarity, after controlling for all the potential confounding factors (model II), the relationship between the two was still present. The effect size of the group < 6 h and ≥ 8 h were (β, 3.58 [95% CI 1.60, 5.56]), (β, 1.70 [95% CI 0.76, 2.64]), respectively. In terms of DBP, the results of multiple linear regression analysis were illuminated in supplementary table 1. After controlling all the potential confounding factors, the significant association was not founded in sleep duration < 6 h (β, 0.28[95% CI − 1.25, 1.82]) and ≥ 8 h(β, − 0.41[95% CI − 1.14, 0.32]).

**Table 3:** Relationship between sleep duration and SBP in different models.

Exposure	Crude model (β, 95% CI, P)	Model I (β, 95% CI, P)	Model II (β, 95% CI, P)
**Sleep duration**			
6–8 h	Ref	Ref	Ref
< 6 h	6.15 (3.88, 8.42) < 0.0001	4.17 (2.19, 6.15) < 0.0001	3.58 (1.60, 5.56) 0.0004
≥ 8 h	0.11 (-1.00, 1.21) 0.8513	1.55 (0.60, 2.51) 0.0015	1.70 (0.76, 2.64) 0.0004

Crude model adjust for: None; Model I adjust for: Gender; Age; Race; Model II adjust for: Gender; Age; Race; alcohol; Albumin; Creatinine; Hemoglobin; diabetes; hypertension; snort or stop breathing; smoke; TC; BMI; AST; HDL.

*CI* confidence interval, *Ref* reference, *TC* total cholesterol, *BMI* body mass index, *AST* aspertate aminotransferase, *HDL* high-density lipoprotein, *SBP* systolic blood pressure.

### Subgroup analyses of factors influencing the association between sleep duration and SBP

In the subgroup analysis stratified by gender, age and race, the association between sleep duration and SBP was explored in Table 4. The positive effect was evident in most subgroups. All the potential confounding factors except the subgroup variable were adjusted. It showed that in females, sleep duration (< 6 h, ≥ 8 h) on workdays was associated with SBP (β = 5.99, 2.41, respectively, all *P* < 0.0005). Moreover, the association was much more obvious among participants aged (≥ 60) with sleep duration < 6 h. The effect size was 7.23 (*P* = 0.0217). In the subgroup classified by race, a significantly positive association was found in White whose sleep duration < 6 h or ≥ 8 h (β = 6.64 (*P* = 0.0007), and 1.91 (*P* = 0.0215), respectively). In others race, sleep duration (≥ 8 h) was associated with SBP (β = 2.06, *P* = 0.0097).

**Table 4:** Effect size of sleep duration on SBP in each subgroup.

Characteristic	N	6–8 h	< 6 h (β, 95% CI, P)	≥ 8 h (β, 95% CI, P)
**Gender**				
Male	1378	Ref	1.22 (− 1.31, 3.76) 0.3440	0.92 (− 0.39, 2.23) 0.1685
Female	1509	Ref	5.99 (2.93, 9.04) 0.0001	2.41 (1.07, 3.75) 0.0004
**Age**				
< 45	1884	Ref	1.18 (− 0.85, 3.20) 0.2543	0.74 (− 0.16, 1.65) 0.1082
≥ 45, < 60	495	Ref	4.80 (− 0.72, 10.32) 0.0890	0.21 (− 2.44, 2.86) 0.8777
≥ 60	508	Ref	7.23 (1.08, 13.38) 0.0217	1.86 (− 1.60, 5.32) 0.2920
**Race**				
Mexican American	302	Ref	2.09 (− 4.11, 8.30) 0.5096	− 0.38 (− 2.87, 2.11) 0.7649
White	968	Ref	6.64 (2.82, 10.45) 0.0007	1.91 (0.28, 3.54) 0.0215
Black	550	Ref	− 0.76 (− 4.12, 2.60) 0.6587	1.04 (− 1.41, 3.49) 0.4063
Others	1067	Ref	2.95 (− 0.24, 6.13) 0.0699	2.06 (0.50, 3.61) 0.0097

Adjusted for: Gender; Age; Race; alcohol; Albumin; Creatinine; Hemoglobin; diabetes; hypertension; snort or stop breathing; smoke; TC; BMI; AST; HDL except the subgroup variable.

*CI* confidence interval, *Ref* reference, *TC* total cholesterol, *BMI* body mass index, *AST* aspertate aminotransferase, *HDL* high-density lipoprotein.

## Discussion

The sleep duration of the general population has been affected by modern life, which also has been an important public health issue that has attracted the attention of researchers. Previous studies proved that sleep duration might also contribute to the increase in blood pressure^10–12,15^. We found sleep duration in non-overweight/obese people positively correlated with SBP. In females, sleep duration < 6 h or ≥ 8 h on workdays were associated with SBP. In middle and old age, insufficient sleep duration (< 6 h) can lead to higher levels of SBP. In comparison with other ethnic groups, sleep duration < 6 h or ≥ 8 h was also associated with higher SBP among the White population.

A lot of cross-sectional and longitudinal epidemiological studies were used to explore the connection among sleep duration and hypertension. In the 2007–2009 National Healthy Interview Surveys (NHIS)(n = 71,455), compared with the 8-h group, adults who slept for less than 6 h or 6 h were more likely to develop hypertension (odds ratio (OR): 1.49 (1.34–1.64) and 1.15 (1.08–1.23), respectively)^11^. Several meta-analysis researches confirmed that short sleep duration (≤ 5 h or ≤ 6 h) was associated with hypertension, but there was no evidence of heterogeneity^19,20^. However, few studies supported that short sleep duration had no impact on hypertension^21^.

As to long sleep duration, the relationship was not very clear but increasingly of interest. One sleep heart health study with 5910 participants discovered that compared to the 7–8 h of sleep duration, the adjusted OR of 8–9 h and ≥ 9 h of sleep duration to hypertension were 1.19 (1.04–1.37) and 1.30 (1.04–1.62), respectively^22^. The data of 71,455 participants from 2007 to 2009 NHIS also demonstrated that sleep ≥ 10 h was associated with a 20% higher risk of hypertension than sleep of 8 hours^11^. But, two meta-analyses showed that no significant relationship was found between long sleep duration and the occurrence of hypertension^9,20^.

The biological mechanisms underlying the association of sleep duration with blood pressure isn’t clear. Sleep duration, especially short sleep, may affect BP, by increasing sympathetic nerve excitement, reducing parasympathetic nerve activity^8^. These changes in autonomic nerve tension will lead to faster heart rate, increased heart rate orthostatic reactivity, and decreased high-frequency heart rate variability^23^. Short sleep duration means sleeping too late or getting up too early. It also means being exposed to light much longer. Light transmits non-image forming function light information to the brain through retinal ganglion cells, such as sleep–wake and circadian rhythm regulation^24^. So short sleep duration will disrupt 24 h sleep–wake cycle, which is an integrated process involving rhythmic changes in endocrine, autonomy, movement, sensation and brain activity. This change will inevitably have a certain impact on blood pressure^25^. Research have shown that when a person’s sleep–wake cycle is inconsistent with the external environment, the average arterial pressure will increase by 3%^26^. The inflammatory process may also play a vital role in the pathogenesis and pathophysiology of the relationship between short or long sleep duration and BP^27^. Inflammatory factors such as c-reactive protein and interleukin-6 is increased with prolonged sleep duration, which can cause drowsiness and fatigue, and may also increase the risk of hypertension in people with long sleep duration^27^. Another study showed that short sleep duration was associated with elevated C-reactive protein level^28^. Long sleep duration was also associated with an increased risk of obesity, metabolic syndrome, and type 2 diabetes^7^.The underlying mechanisms were that long sleep duration could disrupt circadian clocks and decrease insulin sensitivity, leading to unhealthy eating habits, decreased calorie consumption, and elevated systemic inflammation^29^. Additionally, long sleep duration has been identified to be related to sleep fragments, which could activate sympathetic nervous system and lead to increased BP^30,31^.

Our research can bring some inspiration to clinical work. For example, for non-overweight/obese female patients with borderline hypertension or poor blood pressure control, we can improve SBP by adjusting sleep time. We need to pay more attention to the impact of sleep duration on SBP among non-overweight/obese people, especially in regards to females and the elderly.

Like most studies, our study also has some shortcomings. Firstly, sleep duration was only based on subjective evaluation. However, subjective sleep time and objective sleep time were only moderately correlated, and there might be a certain bias^32^. Secondly, another issue to consider was the lack of a consistent standard for long and short sleep duration. At last, we did not consider other potential confounding factors, such as sleep quality, the use of sleeping pills, socio-economic status and educational status. Further studies with more variables and larger populations should be done for validating our results.

## Conclusions

In non-overweight/obese population, especially in females, both short sleep duration(< 6 h) and long sleep duration(≥ 8 h) on workdays were correlated with higher levels of SBP. In old age populations, insufficient sleep duration (< 6 h) was associated with higher levels of SBP. Compared with other ethnic groups, sleep duration (< 6 h or ≥ 8 h) was also associated with higher SBP in Whites.

## Supplementary Information

Supplementary Table 1.

## Data Availability

The datasets used and/or analyzed during the present study were availed by the corresponding author on reasonable request.

## Abbreviations

SBP: Systolic blood pressure
DBP: Diastolic blood pressure
BP: Blood pressure
TC: Total cholesterol
BMI: Body mass index
AST: Aspertate aminotransferase
HDL: High-density lipoprotein
CI: Confidence interval
OR: Odds ratio

## References

[1] Hunter DJ (2005). Gene-environment interactions in human diseases. Nat. Rev. Genet..

[2] Yoon SS, Ostchega Y, Louis T (2010). Recent trends in the prevalence of high blood pressure and its treatment and control, 1999–2008. NCHS Data Brief.

[3] Pereira M, Lunet N, Azevedo A, Barros H (2009). Differences in prevalence, awareness, treatment and control of hypertension between developing and developed countries. J. Hypertens..

[4] Antikainen RL, Moltchanov VA, Chukwuma CC (2006). Trends in the prevalence, awareness, treatment and control of hypertension: The WHO MONICA Project. Eur J Cardiovasc Prev Rehabil.

[5] Egan BM, Zhao Y, Axon RN (2010). US trends in prevalence, awareness, treatment, and control of hypertension, 1988–2008. JAMA.

[6] Forouzanfar MH, Liu P, Roth GA (2017). Global burden of hypertension and systolic blood pressure of at least 110 to 115 mm Hg, 1990–2015. JAMA.

[7] St-Onge MP, Grandner MA, Brown D (2016). Sleep duration and quality: impact on lifestyle behaviors and cardiometabolic health: A Scientific Statement From the American Heart Association. Circulation.

[8] Gangwisch JE (2014). A review of evidence for the link between sleep duration and hypertension. Am. J. Hypertens..

[9] Meng L, Zheng Y, Hui R (2013). The relationship of sleep duration and insomnia to risk of hypertension incidence: A meta-analysis of prospective cohort studies. Hypertens. Res..

[10] Cappuccio FP, Stranges S, Kandala NB (2007). Gender-specific associations of short sleep duration with prevalent and incident hypertension: The Whitehall II Study. Hypertension.

[11] Fang J, Wheaton AG, Keenan NL, Greenlund KJ, Perry GS, Croft JB (2012). Association of sleep duration and hypertension among US adults varies by age and sex. Am. J. Hypertens..

[12] Kim J, Jo I (2010). Age-dependent association between sleep duration and hypertension in the adult Korean population. Am. J. Hypertens..

[13] Fernandez-Mendoza J, Vgontzas AN, Liao D (2012). Insomnia with objective short sleep duration and incident hypertension: The Penn State Cohort. Hypertension.

[14] Lopez-Garcia E, Faubel R, Guallar-Castillon P, Leon-Muñoz L, Banegas JR, Rodriguez-Artalejo F (2009). Self-reported sleep duration and hypertension in older Spanish adults. J. Am. Geriatr. Soc..

[15] Faraut B, Touchette E, Gamble H (2012). Short sleep duration and increased risk of hypertension: A primary care medicine investigation. J. Hypertens..

[16] Gangwisch JE, Heymsfield SB, Boden-Albala B (2006). Short sleep duration as a risk factor for hypertension: Analyses of the first National Health and Nutrition Examination Survey. Hypertension.

[17] Dua S, Bhuker M, Sharma P, Dhall M, Kapoor S (2014). Body mass index relates to blood pressure among adults. N. Am. J. Med. Sci..

[18] Tesfaye F, Nawi NG, Van Minh H (2007). Association between body mass index and blood pressure across three populations in Africa and Asia. J. Hum. Hypertens..

[19] Obesity: preventing and managing the global epidemic. Report of a WHO consultation. World Health Organ Tech Rep Ser. 2000. 894: i–xii, 1–253. 11234459

[20] Guo X, Zheng L, Wang J (2013). Epidemiological evidence for the link between sleep duration and high blood pressure: A systematic review and meta-analysis. Sleep Med..

[21] Matsumoto T, Murase K, Tabara Y (2018). Impact of sleep characteristics and obesity on diabetes and hypertension across genders and menopausal status: The Nagahama study. Sleep.

[22] Gottlieb DJ, Redline S, Nieto FJ (2006). Association of usual sleep duration with hypertension: The Sleep Heart Health Study. Sleep.

[23] Castro-Diehl C, Diez Roux AV, Redline S (2016). Sleep duration and quality in relation to autonomic nervous system measures: The Multi-Ethnic Study of Atherosclerosis (MESA). Sleep.

[24] Prayag AS, Münch M, Aeschbach D, Chellappa SL, Gronfier C (2019). Light modulation of human clocks, wake, and sleep. Clocks Sleep.

[25] Smolensky MH, Hermida RC, Castriotta RJ, Portaluppi F (2007). Role of sleep-wake cycle on blood pressure circadian rhythms and hypertension. Sleep Med..

[26] Scheer FA, Hilton MF, Mantzoros CS, Shea SA (2009). Adverse metabolic and cardiovascular consequences of circadian misalignment. Proc. Natl. Acad. Sci. USA.

[27] Patel SR, Zhu X, Storfer-Isser A (2009). Sleep duration and biomarkers of inflammation. Sleep.

[28] Chiang JK (2014). Short duration of sleep is associated with elevated high-sensitivity C-reactive protein level in Taiwanese adults: a cross-sectional study. J. Clin. Sleep Med..

[29] Tan X, Chapman CD, Cedernaes J, Benedict C (2018). Association between long sleep duration and increased risk of obesity and type 2 diabetes: A review of possible mechanisms. Sleep Med. Rev..

[30] Grandner MA, Drummond SP (2007). Who are the long sleepers? Towards an understanding of the mortality relationship. Sleep Med. Rev..

[31] Stamatakis KA, Punjabi NM (2010). Effects of sleep fragmentation on glucose metabolism in normal subjects. Chest.

[32] Lauderdale DS, Knutson KL, Yan LL, Liu K, Rathouz PJ (2008). Self-reported and measured sleep duration: How similar are they. Epidemiology.

