# Supplementary material for: Association between sleep duration on workdays and blood pressure in non-overweight/obese population in NHANES: a public database research
Source: Sci Rep. 2022 Jan 21;12:1133. doi: 10.1038/s41598-022-05124-y (PMC8782988; doi:10.1038/s41598-022-05124-y)
Supplement: Supplementary file 1 — Supplementary Table 1. [file 41598_2022_5124_MOESM1_ESM.docx]

**Supplementary Table 1. Relationship between sleep duration and DBP in different models**

| Exposure | Crude model (β,95%CI, P) | Model I (β,95%CI, P) | Model II (β,95%CI, P) |
| --- | --- | --- | --- |
| Sleep duration |  |  |  |
| 6-8 hours | Ref | Ref | Ref |
| <6 hours | 1.25 (-0.32, 2.83) 0.1195 | 0.70 (-0.84, 2.24) 0.3759 | 0.28 (-1.25, 1.82) 0.7166 |
| ≥8 hours | -1.09 (-1.86, -0.33) 0.0052 | -0.58 (-1.33, 0.17) 0.1280 | -0.41 (-1.14, 0.32) 0.2740 |

**Abbreviations:** CI, confidence interval; Ref, reference; TC，total cholesterol；BMI，body mass index; AST, aspertate aminotransferase; HDL, high-density lipoprotein; DBP, diastolic blood pressure.

Crude model adjust for: None；

Model I adjust for: Gender; Age; Race；

Model II adjust for: Gender; Age; Race; alcohol; Albumin; Creatinine; Hemoglobin; diabetes; hypertension; snort or stop breathing; smoke; TC; BMI; AST; HDL.
